# Supplementary material for: Identification of photoperiod-induced specific miRNAs in the adrenal glands of Sunite sheep (Ovis aries)
Source: Front Vet Sci. 2022 Jul 22;9:888207. doi: 10.3389/fvets.2022.888207 (PMC9354845; doi:10.3389/fvets.2022.888207)
Supplement: Supplementary file 1 [file Data_Sheet_1.ZIP › Supplementary file/miRDeep2 file.zip!/miRDeep2 file/Novel_Structure/SP42A3/Novel_185.pdf]

Provisional ID	:	NC_019484.2_32008
Score total	:	2
Score for star read(s)	:	-1.3
Score for read counts	:	0
Score for mfe	:	1.7
Score for randfold	:	1.6
Score for cons. seed	:	
Total read count	:	119
Mature read count	:	119
Loop read count	:	0
Star read count	:	0

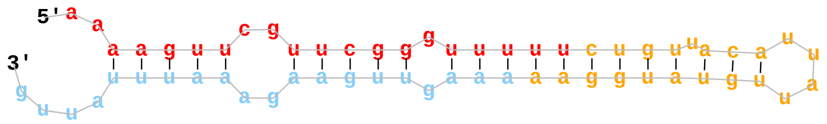

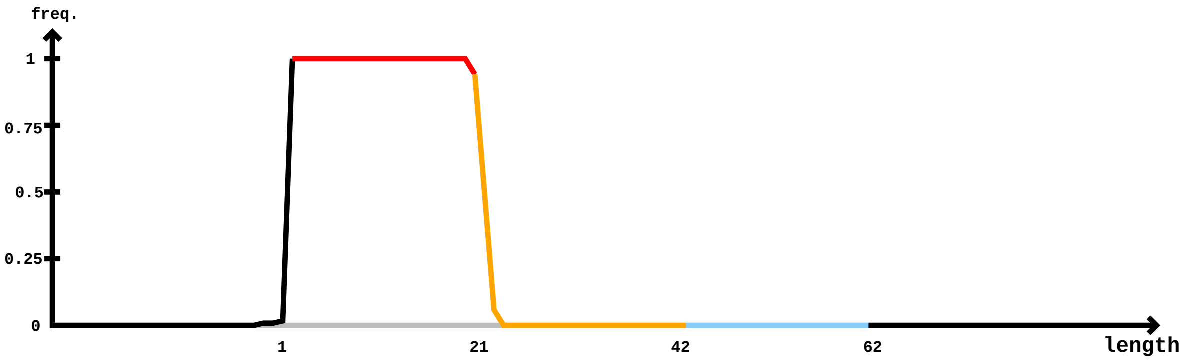

Mature **Star**

```
5'- aucuugaccuauuggguuggugaaaaaguucguucggguuuuucuguuacauuauuguauggaaaaaguugaagaaauuuauuggccaacccaguacaacuuuggugcuuuuc   -3'    exp
      ......((((((((((((((.((.(((((..(((((.(((((((((.(((....)))))))))))))).))))).)))))..))))).)).)))))))))))).......))).......   reads      mm        sample
      ....................ugaaaaaguucguucggguuuuuu.................................................................   1          0         seq
      ......................aaaaaguuUguucggguuuuuu.................................................................   1          1         seq
      .......................aaaaguucguucggguuuu...................................................................   7          0         seq
      .......................aaaaguucguucggguuuuuu.................................................................   104        0         seq
      .......................aaaaguucguucggguuuuuucu...............................................................   7          0         seq
```
